# Standard and variant Philadelphia translocation in a CML patient with different sensitivity to imatinib therapy^☆^

**DOI:** 10.1016/j.lrr.2013.07.004

**Published:** 2013-08-31

**Authors:** Stefania Aliano, Gabriella Cirmena, Giuseppina Fugazza, Roberto Bruzzone, Claudia Palermo, Mario Sessarego

**Affiliations:** Department of Internal Medicine, I.R.C.C.S. A.O.U. San Martino—IST, Genoa, Italy

**Keywords:** CML, Variant translocation, BCR/ABL KD mutation, Tyrosine kinase inhibitors

## Abstract

Most chronic myeloid leukemia (CML) patients show the Philadelphia chromosome (Ph) arising from the reciprocal t(9;22), but 5–10% present variants of this translocation involving different breakpoints besides 9q34 and 22q11.

We report the non simultaneous occurrence of two different types of Ph translocation in a CML patient: a t(9;22)(q34;q11) standard and a three-way variant t(9;11;22)(q34;p15;q11).

Bone marrow cells with standard translocation did not have BCR/ABL kinase domain (KD) mutations and were sensitive to imatinib therapy. In contrast, bone marrow cells with the variant translocation showed two BCR/ABL KD mutations and were resistant to imatinib, thus inducing transformation to the blast phase and karyotype evolution.

## 1 Case report

A 56-year-old male was admitted to our Hospital in October 2010 because of asthenia, slight fever, and weight loss. Blood examination revealed hemoglobin 7.5 g/dL, platelet count 495,000/uL, white blood cell count 335,000/uL with neutrophils 80%, basophils 10%, myelocytes 6%, and promyelocytes 3%. Lactate dehydrogenase was 1440 U/L. Physical examination revealed splenomegaly. Bone marrow (BM) aspirate showed myeloid hyperplasia. The hematological picture was indicative of accelerated phase chronic myeloid leukemia (CML).

**Fig. 1:**
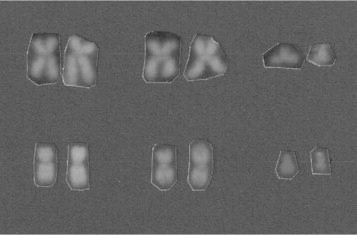
Q-banded karyotype. *Upper*: partial Q-banded karyotype showing the chromosomes 9 and 22 involved in the t(9;22)(q34;q11) as compared with the chromosomes 11 that were not involved. *Lower*: partial Q-banded karyotype showing the chromosomes 11 and 22 involved in the t(11;22)(p15;q11) as compared with chromosomes 9 that were apparently not involved.

**Fig. 2:**
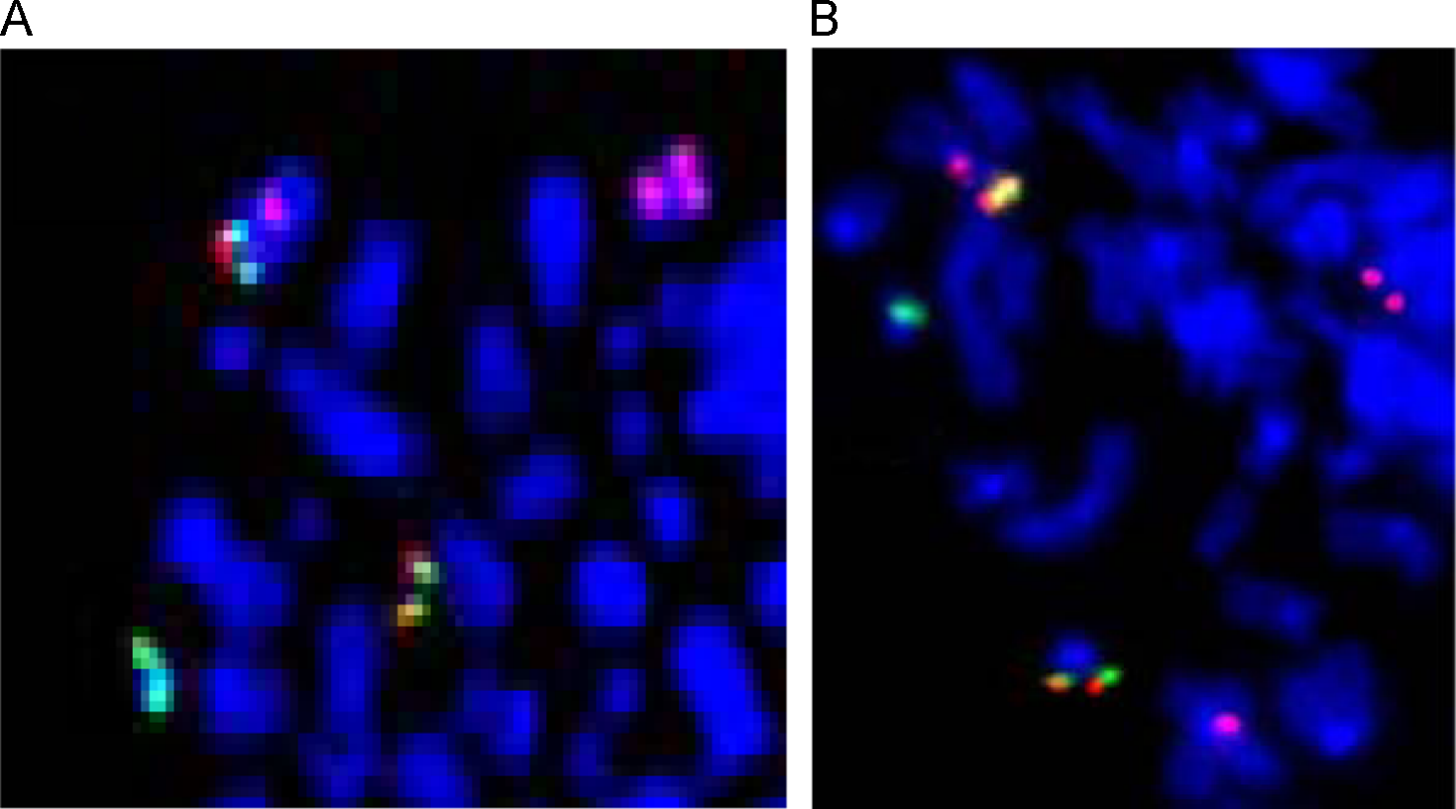
Fluorescent in situ hybridization. (A) FISH on partial metaphase with t(9;22)(q34;q11) using BCR/ABL Dual Color Dual Fusion translocation, Vysis(D-FISH) and Human Chromosome 9 Centromeric Cambio probe (20 metaphases and 200 cells analyzed). *D-FISH probe*: fusion signal (yellow) was present on Philadelphia chromosome and on the long arm of derivative chromosome 9, one orange signal (ABL probe) was present on the long arm of normal chromosome 9, and one green signal (BCR probe) was present on the long arm of normal chromosome 22. *Human Chromosome 9 Centromeric Cambio probe*: two red signals were present on normal and derivative chromosome 9. (B) FISH on partial metaphase with t(11;22)(p15;q11) using BCR/ABL Dual Color Dual Fusion translocation, Vysis (D-FISH) and Human Chromosome 11 Centromeric Cambio probe (20 metaphases and 200 cells analyzed). *D-FISH probe*: fusion signal (yellow) was present on der(22) and on the short arm of derivative chromosome 11, one orange signal (ABL probe) was present on the long arm of normal chromosome 9, and one green signal (BCR probe) was present on the long arm of normal chromosome 22. *Human Chromosome 11 Centromeric Cambio probe*: two red signals were present on normal and derivative chromosome 11. (For interpretation of the references to color in this figure legend, the reader is referred to the web version of this article.)

Cytogenetic analysis showed the presence of a t(9;22)(q34;q11) in all examined metaphases (Fig. 1 upper). Fluorescent in situ hybridization (FISH) using the LSI BCR/ABL Dual-Color, Dual Fusion (D-FISH) (Abbott Molecular-Vysis, Des Plaines,IL) showed two fusions, one green and one orange signal (2F1G1O) confirming the presence of the BCR/ABL1 and ABL1/BCR fusion genes without deletions adjacent to the translocation junctions (Fig. 2A).

Real-time quantitative polymerase chain reaction (RQ-PCR) was performed to quantify BCR/ABL1 transcript as described previously [1]. The quantitative results were expressed as percent ratios relative to an ABL1 internal control using the following formula (p210 ^BCR–ABL^/ABL1×100×CF) [2]. The level of BCR/ABL1 expression was 31% at diagnosis (IS).

The patient was first treated with hydroxyurea at 3 g/day for 7 days, after which imatinib (Gleevec, Novartis, Basel, Switzerland) was administered at a dosage of 600 mg/day.

In January 2011, cytogenetic analysis revealed an unexpected t(11;22)(p15;q11) with chromosomes 9 apparently not being involved (Fig. 1 lower). D-FISH signal pattern proved to be 2F1G1O, with one fusion signal on the der(22), a second fusion signal on 11p15, and one orange and one green signal on normal chromosomes 9 and 22, respectively (Fig.2B). The karyotype was then interpreted as t(9;11;22)(q34;p15;q11) [3]. BCR/ABL1 expression had increased to 39.5% (IS).

The patient was treated with the ICE chemotherapy protocol consisting of idarubicin 8 mg/m2/day, cytarabine (ARA-C) 800 mg/m^2^ and etoposide 150 mg/m^2^/day.

In March 2011, cytogenetic analysis was performed again and we observed a high number of metaphases: 49 were normal whereas 23 showed Ph due to the classical translocation. No cells carrying the variant translocation were observed.

The patient was treated again with imatinib 800 mg/day without hematological response.

In June 2011, white blood cell count was 28,300/uL with 5% of blasts. The karyotype was: 49,XY,t(9;11;22)(q34;p15;q11),+6,+9,+der(22)t(9;22). No cells carrying the standard translocation were observed, indicating that for the second time the imatinib therapy had suppressed the t(9;22) clone, thus allowing the reappearance of the t(9;11;22) clone.

In July 2011, the patient underwent allogenic bone marrow transplantation (BMT) from his HLA compatible sister: the karyotype was 46,XX and the patient reached and maintained complete hematological and molecular remission until October 2012.

In November 2012, the patient progressed to myeloid blast crisis and the karyotype became: 47,XY,t(9;11;22)(q34;p15;q11),+der(22).

The patient died in December 2012.

## 2 Results

At diagnosis the patient was in accelerated phase CML with standard translocation. After three months of therapy with imatinib, cytogenetic analysis revealed the persistence of Ph in all examined metaphases but with a variant translocation involving a third chromosome, t(9;11;22) which had not been observed in the previous cytogenetic analysis (Fig.1).

ICE-chemotherapy, which proved to be clinically unsatisfactory**,** apparently reduced the variant translocation clone but led to the expansion of the Ph+ clone with standard translocation.

The following cycle of imatinib 800 mg/day before BMT was also ineffective. The variant translocation reappeared with additional chromosomal abnormalities including Ph duplication.

This clone with variant Ph translocation appeared to have been eradicated by BMT (the patient reached complete molecular response with RQ-PCR), however, it reappeared 16 months later causing relapse into the myeloid blastic phase.

Quinacrine-bandend (Q-banded) and FISH techniques revealed the presence of the two Ph translocations (standard and variant) alternatively with ABL1/BCR construct location on 9q34 or 11p15 and without adjacent deletions in the junctions of the BCR and/or ABL1 genes (Fig. 2).

We had never previously observed the simultaneous presence of cells with the standard translocation and others with the variant in the same patient.

**Fig. 3:**
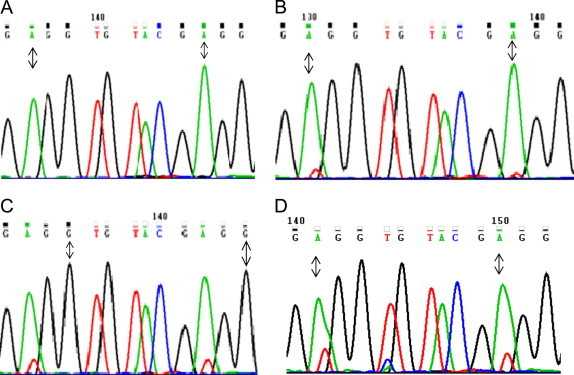
Kinase domain (KD) mutations. Evidence of a BCR/ABL1 kinase domain mutation by direct sequencing (ABL sequence accession number X16416): at diagnosis native BCR–ABL1 (A); detection at 3–8 months of low- level mutation of residues 255E<V and 258 E<V, common nucleotide changes A>T (B, C) and increase of level mutation at 25 months after BMT in myeloid blastic phase. (D).

Considering the alternation of the type of translocation, which would appear to be related to the kind of therapy, we observed that cells carrying the standard translocation were sensitive to imatinib, while the cells bearing the variant translocation were sensitive to ICE chemotherapy. The BM samples that we had stored at diagnosis and after three months of imatinib therapy were therefore screened for kinase domain (KD) mutations in the BCR–ABL1 gene using direct sequencing as described previously [4]. The BM sample taken at diagnosis showing the standard translocation had no detectable BCR–ABL1 KD mutation (Table 1, Fig. 3A), while two different KD mutations were identified in the sample carrying the variant translocation: the first mutation has already been reported and was at codon position 255 (E255V), while the second mutation, which has never previously been described, was at position 258 (E258V) (Table 1, Fig. 3B–D) [5]. The two mutations led to an A to T transition that resulted in a glutamic acid to valine substitution in the ABL1 protein and both mutations should be present at higher than 20%.

These point-mutations in the tyrosine kinase domain of BCR–ABL1 may cause progressive clinical resistance to imatinib and are associated with a greater likelihood of progression to blast crisis and shorter survival [6].

## 3 Discussion

Our case presents two points of interest:

1) The presence of both standard and variant Ph translocations in a single patient. It is likely that the variant translocation occurred after the formation of the standard one: ABL1/BCR complex moved from 9q34 band to 11p15 band thereby restoring apparent cytogenetic integrity to the involved chromosome 9. FISH analysis revealed a 2F1G1O signal pattern which is indicative of the two-step mechanism of variant formation [7] without deletions adjacent to the junctions [8]. It is also likely that the variant translocation was already present at diagnosis but in such a low proportion that it could not be detected by banding and FISH techniques.
2) The second observation of interest is the fact that the leukemic cells with standard translocation did not show mutations in BCR/ABL1 KD and that these cells were sensitive to imatinib. On the contrary, the cells with the variant translocation had two KD mutations in the BCR/ABL1 gene i.e., E255V, which is known to confer resistance to imatinib, and E258V, whose biological significance and ability to confer resistance is unknown [5].

The presence of the two KD mutations in cells with the variant translocation alone is not easy to interpret. It seems unlikely that the transposition of the complex ABL1/BCR from 9q34 to 11p15 is correlated with the occurrence of mutations.

Of the mechanisms of resistance to imatinib, point mutations in the ABL1 KD are among the most frequently investigated. Several mutations are known to confer differing levels of resistance to the available tyrosine kinase inhibitors [5].

The modalities of occurrence and/or selection of mutations in the ABL1 KD sequence are under investigation, but some authors believe that the mutations can antedate treatment with imatinib [9]. This theory is in agreement with the hypothesis that the KD mutations in our patient were present in a number of proliferating leukemic cells, and that imatinib rapidly selected the clone bearing the mutations. Establishing the presence of leukemic clones with KD mutations as early on as possible could therefore lead to the timely use of different tyrosine kinase inhibitors [10].

In conclusion, we demonstrated that cells with the standard Ph had no detectable BCR–ABL1 KD mutations and that they were responsive to imatinib therapy, while cells with the variant translocation harbored two KD mutations, a previously described one and a never previously reported one [5]. These cells were resistant to imatinib, and in our case they were associated with the transformation to blast phase with karyotype evolution.

## Figures and Tables

**Table 1:** Karyotypes and Kinase domain (KD) mutations during the course of the disease. Correlation between karyotypes and KD mutations in order to monitor the cytogenetic and molecular changes.

**Months**	**Karyotypes**	**BCR–ABL1 Kinase Domain mutation**
At diagnosis (October 2010)	46,XY,t(9;22)(q34;q11), [20 metaphases]	native BCR–ABL1
3 months (January 2011)	46,XY,t(9;11;22)(q34;p15;q11), [20 metaphases]	low- level mutation: E255V and E258V
5 months (March 2011)	46,XY [49 metaphases]/46,XY,t(9;22)(q34;q11), [23 metaphases]	native BCR–ABL1
8 months (June 2011)	49,XY,t(9;11;22)(q34;p15;q11),+6,+9,+der(22) t(9;22), [20 metaphases]	low- level mutation: E255V and E258V
9 months- 24 months (July 2011–October 2012) after BMT	46,XX, [20 metaphases]	BCR–ABL1 not present
25 months (November 2012)	47,XY,t(9;11;22)(q34;p15;q11),+der(22). [20 metaphases]	increase of level mutation E255V and E258V
